# Molecular Aspects and Future Perspectives of Cytokine-Based Anti-cancer Immunotherapy

**DOI:** 10.3389/fcell.2020.00402

**Published:** 2020-06-03

**Authors:** Daria S. Chulpanova, Kristina V. Kitaeva, Andrew R. Green, Albert A. Rizvanov, Valeriya V. Solovyeva

**Affiliations:** ^1^Institute of Fundamental Medicine and Biology, Kazan Federal University, Kazan, Russia; ^2^Nottingham Breast Cancer Research Centre, Division of Cancer and Stem Cells, School of Medicine, University of Nottingham Biodiscovery Institute, Nottingham, United Kingdom

**Keywords:** cancer immunotherapy, cytokines, anticancer immune responses, immune cell markers/populations, tumor microenvironment

## Abstract

Cytokine-based immunotherapy is a promising field in the cancer treatment, since cytokines, as proteins of the immune system, are able to modulate the host immune response toward cancer cell, as well as directly induce tumor cell death. Since a low dose monotherapy with some cytokines has no significant therapeutic results and a high dose treatment leads to a number of side effects caused by the pleiotropic effect of cytokines, the problem of understanding the influence of cytokines on the immune cells involved in the pro- and anti-tumor immune response remains a pressing one. Immune system cells carry CD makers on their surface which can be used to identify various populations of cells of the immune system that play different roles in pro- and anti-tumor immune responses. This review discusses the functions and specific CD markers of various immune cell populations which are reported to participate in the regulation of the immune response against the tumor. The results of research studies and clinical trials investigating the effect of cytokine therapy on the regulation of immune cell populations and their surface markers are also discussed. Current trends in the development of cancer immunotherapy, as well as the role of cytokines in combination with other therapeutic agents, are also discussed.

## Immune System and Cancer

The immune system is a complex system consisting of immune cells, compliment proteins and cytokines which provide communication and coordinated cell functions. The coordinated functioning of the immune system allows the maintenance of the body’s homeostasis, eliminating foreign antigens or proteins (Eyileten et al., 2016). The immune system can protect the body against virus-induced tumors by limiting or terminating viral infection. Also, the well-timed elimination of pathogens by the immune system and the rapid termination of inflammation prevents the formation of inflammatory sites which are favorable for tumor development. Finally, the immune system can identify and eliminate tumor cells relying on the recognition of tumor-specific antigens. This process of identifying transformed cells and eliminating them before the tumor forms is referred to as immunological surveillance (Terme and Tanchot, 2017). However, recent discoveries in human immunology also confirm the involvement of immune cells in the maintenance of tumor development and growth (Duan et al., 2014).

Depending on speed and specificity of the immune response innate and adoptive immune responses can be identified. Innate immunity is assumed to be rapid but non-specific response mediated by phagocytes (neutrophils, monocytes, and macrophages), natural killer (NK) cells and complement system (Liu and Zeng, 2012). On the contrary, adaptive immune response takes time to mature naive lymphocytes, T- and B cells, into effector T-cells and secreting B cells (Chaplin, 2010).

It is known that innate and adoptive immune responses are involved in surveying tumor development which can be divided into three stages: elimination, equilibrium, and escape (Dunn et al., 2004; O’Sullivan et al., 2012). With elimination, the innate immune system is able to identify and destroy a nascent tumor using a number of inflammatory cells and signaling molecules (Ricciardi et al., 2015). However, if the tumor cell with a rare and aggressive mutation survives at the elimination stage, the process proceeds to the next stage of equilibrium in which excessive growth of the tumor is prevented by immunological mechanisms. At this second equilibrium stage, the tumor cells are not completely destroyed, but the tumor growth is controlled by the immune system (Ricciardi et al., 2015). The equilibrium stage is the longest of the three stages and can last up to 20 years, from the initial transforming event to the clinical manifestation of the tumor (Miliotou and Papadopoulou, 2018). The escape stage occurs when tumor cells which have survived both elimination and equilibrium become resistant to immunological surveillance allowing the tumor to grow and become clinically detectable (Dunn et al., 2004).

Tumors inherently are characterized by low immunogenicity since they arise from the body’s normal cells. The tumor microenvironment (TME), which in addition to the tumor cells contains a large number of other cell populations (Chulpanova et al., 2018b; Kitaeva et al., 2019) inhibits the tumor killing capacities of immune cells (Borst et al., 2018). Thus, there is a close relationship between the tumor and immune system cells, which is the key to understanding the processes that lead to the elimination of the tumor or its progression. The majority of immune system cells and signaling molecules involved in immunological surveillance will be comprehensively discussed in this review. Understanding the role of immune cells expressing specific surface markers typical for cell populations with pro- or anti-tumor properties, and their identification will allow researchers to develop new methods for predicting tumor progression as well as to identify new targets and possible immune system modulation mechanisms for optimal drug selection. In particular, the success of cytokine-based cancer therapies in modulating various populations of immune system cells and the prospects for the further development of this approach for cancer therapy will be discussed.

## NK Cells

Natural killer cells are important participants in the antitumor immunity, as they detect the major histocompatibility complex (MHC) presented on the surface of all nuclear cells of the body. NK cells secrete perforins and granzymes to induce apoptosis of cells that have abnormal or altered MHCI expression (Brodbeck et al., 2014). Typically, NK cells express CD56, in the absence of CD3 (T-cell receptor). Depending on CD56 surface-density expression NK cells can be divided into two populations – CD56^bright^ (or CD56^high^) and CD56^*dim*^ (or CD56^low^) NK cells (Poli et al., 2009).

CD56^low^ NK cells, which also have high expression of CD16 (CD16^high^), exhibit cytotoxic function and contain large amounts of perforin (Angelo et al., 2015). CD56^high^ CD16^±^ NK cells are characterized by low perforin levels and mainly specialize in the production of cytokines, predominately IFN-γ, which is necessary for the maturation of dendritic cells (DCs) (Stabile et al., 2017). TME can significantly affect population distribution and the function of tumor-infiltrating NK cells (TINKs). For example, a high number of CD56^high^ perforin^low^ NK cells are observed in breast and lung cancers compared with normal tissues. High accumulation of CD56^high^ perforin^low^ NK cells is associated with the secretion of specific chemokine (C-X-C motif) ligand 9 (CXCL9) and CXCL10, which support the migration of non-cytotoxic CD56^high^ NK cells in TME (Carrega et al., 2014). The population of CD56^high^ NK cells also prevails among patients within breast, melanoma, colon cancer (Levi et al., 2015), non-small lung cancer and has a pro-angiogenic effect, thereby promoting tumor growth (Bruno et al., 2013). However, CD56^low^ NK cells found in the lymph nodes infiltrated with tumor cells were highly cytotoxic against autologous melanoma (Ali et al., 2014). Probably, tumor-related soluble factors [e.g., interleukin (IL)10, indoleamine-pyrrole 2,3-dioxygenase (IDO), prostaglandin E2 (PGE2)] and TME cells are responsible for phenotypic and functional changes in NK cells (Stabile et al., 2017) and help tumors to recruit NK cells.

Unlike B and T-cells, NK cells do not undergo gene rearrangements to generate the repertoire of cell surface receptors. Instead, they use germline-encoded inhibiting and activating receptors (Carrillo-Bustamante et al., 2016). NK cells possess the ability to distinguish between normal and transformed cells based on the expression of MHCI on the cell surface. MHCI molecules, which are largely expressed in normal cells, bind to the inhibitory receptors on the surface of NK cells, which leads to NK cell inactivation. In addition to aberrant MHCI expression, transformed cells also acquire stress-induced ligands for activating NK cell receptors (Caligiuri, 2008). The most important activating NK cell receptors are natural cytotoxicity receptors (NKp46, NKp30, and NKp44), C-type lectin natural killer group 2D receptor (NKG2D), DNAX accessory molecule 1 (DNAM1) and immunoglobulin-like killer receptors (KIR2DS and KIR3DS) (Martinet and Smyth, 2015). Inhibitory receptors that can bind to human leukocyte antigen (HLA) class I (HLA-I) or HLA-I-like molecules include two different classes: immunoglobulin-like killer receptors (KIR2DL and KIR3DL) and C-type lectin receptors NKG2A/B (Campbell and Purdy, 2011).

In order to avoid an NK cell mediated immune response, tumor cells secrete various immunosuppressive factors that regulate the expression or functional activity of NK cell receptors. For example, the binding of proliferating cell nuclear antigen (PCNA) to the NKp44 receptor leads to activation of the constitutively inactive immunoreceptor tyrosine-based inhibition motif (ITIM) in the cytoplasmic domain of the receptor, which inhibits the cytotoxic function of NK cells (Rosental et al., 2011). Transforming growth factor-β (TGF-β) and IL10 produced by tumor cells and immune cells of TME can inhibit NKG2D expression (Schiavoni et al., 2013). Other TME participants, tumor-associated fibroblasts, can also inhibit the expression of NKp44, NKp30, and DNAM-1 receptors due to PGE2 secretion, which suppress the antitumor activity of NK cells (Balsamo et al., 2009). As expected, the reduced expression of activating receptors, in particular NKG2D, NKp30, NKp46, DNAM1, is associated with poor prognosis in patients with pancreatic cancer, gastric cancer, colorectal cancer and melanoma (Peng et al., 2013; Mirjacic Martinovic et al., 2014). Whilst the increased expression of inhibitory receptors KIR2DL1 and KIR2DL2/3 negatively correlates with the cytotoxicity of NK cells and enhances the melanoma progression (Naumova et al., 2007). Overexpression of the NKG2A inhibitory receptor is also associated with poor prognosis in patients with breast and colorectal cancer (Balsamo et al., 2009).

## Neutrophils

Neutrophils, polymorphonuclear and granulocytic cells, consist approximately 50–70% of the total immune cell population and act as the first responders to infection and injury (Singel and Segal, 2016). Neutrophils mature in the bone marrow and enter the bloodstream as terminally differentiated cells, carrying CD11b, CD66b, CD16, Ly6G, C-X-C chemokine receptor type 2 (CXCR2) on their surface (Coffelt et al., 2016; Lakschevitz et al., 2016). The role of neutrophils in tumor immunity has long remained unclear, since it was thought that neutrophil lifespan is too short to influence cancer progression. However, neutrophil half-life increases from 7 h in normal conditions to 17 h in cancer (Ocana et al., 2017) which may allow them to further contribute to tumor progression. The effect of neutrophils on tumor progression remains controversial, and N1/N2 nomenclature has been proposed, where N1 are neutrophils that support tumor progression, whilst N2 are those that suppress tumor progression (Fridlender et al., 2009). However, this nomenclature seems to be too simplified and there is not enough information regarding different functions and CD markers to identify N1/N2 neutrophils. A previous study declares that neutrophils can lead to tumor regression (Stoppacciaro et al., 1993). However, most of the information suggests that neutrophils promote tumor growth, mainly by the stimulation of angiogenesis (Nozawa et al., 2006; Jablonska et al., 2010). Neutrophils are also able to suppress the immune response against tumor cells including the secretion of nitric oxide synthase (iNOS), or arginase 1 (ARG1) which suppress CD8^+^ T lymphocyte antitumor response (Fridlender et al., 2009). It is also worth noting that several studies have shown neutrophils to have a pro-tumor effect, while in others no effect has been found (Granot et al., 2011; Li Z. et al., 2012).

## Macrophages

Monocytes originate from circulating peripheral blood monocytes and play an important role in maintaining homeostasis (Wynn et al., 2013). Macrophages are traditionally classified into M1 and M2 macrophages depending on their function in the immune system (Mills et al., 2000). Classically activated macrophages (M1) protect the body from external pathogens and destroy tumor cells by secreting the pro-inflammatory cytokines IL6, IL12, IL23, and tumor necrosis factor-α (TNF-α), as well as releasing reactive oxygen/nitrogen species (Atri et al., 2018). Phenotypically, M1 express CD68, CD80, CD86, CD206^low^, and HLA-II DR isotype (HLA-DR)^high^ (Li W. et al., 2012; Bertani et al., 2017).

The alternatively activated macrophages (M2) are involved in the resolution of inflammation by producing anti-inflammatory IL10 and TGF-β (Aras and Zaidi, 2017). M2 macrophages are positive for CD163 (Hu et al., 2017), CD68, CD206, negative for CD80 and have a low level of HLA-DR expression (Bertani et al., 2017). However, it is worth noting that such a classification is regarded as too simplified, and now the M1< –…– >M2 spectrum concept is considered to be more preferable. According to this concept, macrophages do not differentiate strictly into stable M1 and M2 subsets, but form complex mixed phenotypes depending on the combination of received external stimuli [discussed in detail in Murray et al. (2014)].

Tumor-associated macrophages (TAMs) are close to M2-polarized type. The cells of tumor and TME secrete CCL2 and macrophage colony-stimulating factor (M-CSF) (Qian et al., 2011) and attract a large number of inflammatory monocytes to tumor sites where they differentiate into TAMs due to secretion of IL4, IL10, and IL13 (Wang and Joyce, 2010; Chuang et al., 2016). TAMs can be identified by CD163, CD200R, CD204, CD206, and HLA-DR^low^ expression (Yang et al., 2015; Cope et al., 2016; Kubota et al., 2017). The recruited macrophages represent a significant part of the TME and provide significant support to the tumor. TAMs secrete pro-angiogenic factors, such as vascular endothelial growth factor (VEGF) family members, and provoke neovascularization and lymphovascularisation of the tumor (Linde et al., 2012; Werchau et al., 2012). The secretion of matrix metalloproteinases (MMPs) and cathepsins supports tumor cell invasion and metastasis (Gocheva et al., 2010; He et al., 2016). TAMs are also able to inhibit the CD8^+^ T-cell immune response through direct interaction with T-cells, for example, using programmed cell death 1 ligand 1 (PD-L1) (Kubota et al., 2017) or by secretion of immunosuppressive molecules, such as IL10, TGF-β, ARG1, and PGEs (Kubota et al., 2017; Takahashi et al., 2017).

## Gamma-Delta (γδ) T-Cells

T lymphocytes can be divided into two subtypes: αβ and γδ T-cells. αβ T-cells represent up to 95% of the CD3^+^ cell population and recognize the antigen presented on MHCI or MHCII (Zou et al., 2017) (since αβ T-cells is a larger population, we will use “T-cells” to denote αβ T lymphocytes). The γδ T-cells form the remaining T lymphocyte population, but do not need conventional MHC-dependent antigen presentation (Tanaka et al., 1995). Most often, γδ T-cells are classified by the type of variable chains (γ and δ) in the T-cell receptor (TCR). The vast majority of γδ T lymphocytes (75%) in peripheral-blood express Vδ2 chain and co-express Vγ9 chain (Dimova et al., 2015). Such Vγ9Vδ2 T-cells are able to directly lyse tumor cells due to the secretion of perforin/granzymes (Mattarollo et al., 2007). They can also induce apoptosis of tumor cells through the TNF-related apoptosis-inducing ligand (TRAIL)/TRAIL receptor (TRAILR) system (Zou et al., 2017). Such cytotoxic Vγ9Vδ2 T-cells can be identified by increased NKG2D (natural killer group 2D) (Todaro et al., 2009), CD56 (Alexander et al., 2008), and CD16 expression (Lafont et al., 2001). However, Vγ9Vδ2 T-cells may also have antigen-presenting cell-like activity, which is accompanied by the expression of CD86, CD80, CD40, and MHCII (Pang et al., 2012). Other T-cells that have a Vδ1 chain prevail in tissues and are able to recognize malignant epithelial cells (Maeurer et al., 1996). Both subgroups express CD6 (Zhou et al., 2012). Both subgroups can also be classified by the expression of CD45RA and CD27 on CD45RA^+^ CD27^+^ naïve, CD45RA^–^ CD27^+^ central memory, CD45RA^–^ CD27^–^ effector memory and CD45RA^+^ CD27^–^ effector memory T cells [for more information see Pang et al. (2012)]. However, in TME, under the influence of secreted factors, γδ T-cells can be polarized; i.e., can shift from one phenotype to another, in forkhead box P3 (FOXP3)^+^ regulatory γδ T-cells (γδ Tregs) which display regulatory/immunosuppressive activity (Casetti et al., 2009) or in CD30^+^ γδ T17 cells, which secrete large amounts of IL17 and can provide the accumulation of immunosuppressive cells in the tumor and stimulate angiogenesis (Wu et al., 2014; Patil et al., 2016).

## T-Cells

T-cells with αβ TCR are the main participants in the adaptive immune response and are usually classified by polarization (functional subtypes) or degree of differentiation (naïve, activated, memory) (Geginat et al., 2014). T-cells can be grouped into functional subsets depending on the expression of CD markers. In the process of maturation in the thymus, T-cells acquire CD4 or CD8 markers (Kurd and Robey, 2016). CD4^+^ T-cells (helper T-cells) are divided into different subsets: Th (T helper) 1, Th2, Th9, Th17, Th22, Tregs (regulatory T cells), and Tfh (follicular helper T-cells), which are characterized by different CD expression profiles and their functions in the immune system (Raphael et al., 2015). Th1 can enhance the priming and expansion of CD8 T-cells or inhibit angiogenesis in IFN-γ-dependent manner (Qin and Blankenstein, 2000; Chraa et al., 2019). Most Th1 cells are CXCR3^+^ and CCR4^–^, and also express CCR2, CCR5 and CXCR6 in the activated state (Kim et al., 2001) (activation and memory states are discussed below).

The effect of Th2 on tumor growth is not so clear. IL10, expressed by Th2, inhibits the processing and presentation of antigens by DCs (Steinbrink et al., 2002) and also activates Tregs (Levings et al., 2002). At the same time, another Th2 cytokine IL4 increases tumor infiltration with eosinophils and macrophages that can kill tumor cells (Tepper et al., 1992). Almost all Th2 cells are CCR4^+^ CXCR3^–^ (Kim et al., 2001), and can also express CCR3 (Sallusto et al., 1997) and CCR8 on their surface (Zingoni et al., 1998).

Th17 cells are found in a large number of different tumors, but their effect remains controversial (Asadzadeh et al., 2017). On one hand, Th17 cells are able to secrete various cytokines and chemokines, such as IL17, IL23, CCL20 which promote tumor growth (Tartour et al., 1999; Langowski et al., 2006). On the other hand, IL17 supports the recruitment of NK cells (Lv et al., 2011) and stimulates the production of cytokines by stromal cells, which finally results in the recruitment and activation of neutrophils (Witowski et al., 2000). The interaction of Th17 cells and the tumor is comprehensively discussed in Asadzadeh et al. (2017). Basically, Th17 cells are determined by the CCR6^+^ marker, then two populations of CCR6^+^ Th17 cells can de distinguished. CCR6^+^ CCR4^+^ Th17 cells produce more IL17 as well as IL22 (Acosta-Rodriguez et al., 2007b) and are able to suppress the activity of CD8^+^ T-cells (Greten et al., 2012). Another population, CCR6^+^ CXCR3^+^ Th17 cells, produce less IL17, but also synthesizes IFN-γ (Acosta-Rodriguez et al., 2007b). Th17 cells have also increased expression of IL1, IL6, and IL23 receptors, which are necessary for their differentiation (Acosta-Rodriguez et al., 2007a; Chung et al., 2009).

Tfh are characterized by expression of CD28, CD40L, CXCR5 (or CD185), inducible T-cell costimulator (ICOS), and programmed cell-death protein 1 (PD-1) (Deenick and Ma, 2011; Ame-Thomas et al., 2012) which appear during the process of Tfh differentiation after stimulation by DCs. Tfh cells seem to support antitumor immunity (Jia et al., 2015). Their main function is to promote B cells differentiation into antibody-secreting cells in secondary lymphoid organs (Kim and Cantor, 2014). Tfh cells also secrete IL21, which can stimulate CD8^+^ T-cells (Shi W. et al., 2018). However, Tfh can stimulate growth and survival of some lymphoid tumors. For example, these cells can promote the proliferation of chronic lymphocytic leukemia (CLL) from secondary lymphoid tissue (Pascutti et al., 2013), support follicular lymphoma (FL) (Ame-Thomas et al., 2012).

Tregs are one of the most common T-cell phenotypes in TME (20–30%) (Quezada et al., 2006). They can be detected by the expression of CD25 and FOXP3 transcription factor (Sharma et al., 2005). Tumor cells recruit Tregs from lymphoid organs (Malchow et al., 2013) by the expression of specific chemokines such as CCL2 or CCL21 (Curiel et al., 2004; Shields et al., 2010). Tregs in turn provide suppression of antitumor immunity through the increased expression of PD-1 and cytotoxic T-lymphocyte (CTL)-associated antigen 4 (CTLA-4) (Zappasodi et al., 2018). A number of studies have also been shown that CD39^+^ and/or CD73^+^ Tregs have higher immunosuppressive properties due to the synthesis of adenosine (Borsellino et al., 2007; Deaglio et al., 2007).

Th9 cells are another T-cell population that produces a large amount of IL9 and plays an important role in the antitumor immune response (Rivera Vargas et al., 2017). Th9 cells can both directly lyse tumor cells due to the secretion of granzyme B (GrzmB) (Purwar et al., 2012), and induce TRAIL-mediated apoptosis (Fang et al., 2015). Indirect antitumor activity is mediated by the regulation of DCs and CD8^+^ T-cells which is discussed in detail in Rivera Vargas et al. (2017). Th9 cells express functional CCR3, CCR6, CXCR3 (Kara et al., 2013), and IL17B receptors as well as secrete IL4, TGF-β essential for their differentiation (Veldhoen et al., 2008; Angkasekwinai et al., 2010). Also, since IL1β can induce secretion of IL9 in Th9, thereby increasing antitumor activity (Vegran et al., 2014), it can be concluded that IL1β receptors have been also presented on the surface of Th9.

CD8^+^ T-cells are effector group of T-cells that can kill tumor cells by granule exocytosis and Fas ligand (FasL) (CD95)-mediated apoptosis (Farhood et al., 2019). Activation of naïve CD8^+^ T-cells begins with the binding of CD3 on the T-cell surface with the MHCI-protein complex on the surface of antigen presenting cell (APC). In addition, CD28 replaced on the surface of CD8^+^ T-cell recognizes co-stimulatory B7 proteins (CD80 and CD86) of APC (usually DCs), and CXCR3 expressed in T-cell binds to CXCL9 and CXCL10 chemokines produced by DC (Spranger and Gajewski, 2018). Having bonded with DCs, CD8^+^ T-cells begin to express lymphocyte-function-associated protein 1 (LFA-1) adhesion molecules to enhance binding (Semmrich et al., 2005). After the antigen presentation, CD8^+^ T-cells begin to secrete IL2 and also express its receptor (CD25), thus stimulating proliferation by themselves (Cheng et al., 2002). Following IL2 stimulation, activated CD8^+^ T-cells begin to express lysosomal-associated membrane protein 1 (LAMP-1 or CD107a) which is related to T-cell cytotoxicity (Aktas et al., 2009). Hours or days after activation, CD8^+^ T-cells start expressing PD-1 and CTLA-4, which can form part of a mechanism by which cancer cells would suppress immune responses (Topalian et al., 2016). The relationships of CD8^+^ T-cells and tumors is discussed in detail in Farhood et al. (2019).

Another approach to classify T-cells is by the degree of differentiation. In the process of the differentiation mature, naïve carrying TCR T-cells emerge from the thymus and are able to differentiate into effector and memory cells (Restifo and Gattinoni, 2013). At different stages of differentiation, T-cells carry many different markers on their surface. From the perspective of this article we consider surface markers that are essential for CD-based identification of T-cell populations *in vitro*, but provide links to articles that address these issues in more detail. Naïve T-cells (CD45RA^+^ CD45RO^–^) carry on their surface CD27, CD28 which are essential for interaction with APC (Chen and Flies, 2013). Naïve T-cells are also CCR7^+^ and CD62L^+^ (l-selectin), which ensures their ability to migrate toward secondary lymphoid organs (Picker et al., 1993; Campbell et al., 2001). After antigen presentation, T-cells acquire an effector phenotype (activation) and migrate to the sites of tumor localization. Activated T-cells lose almost all CD markers of naïve T-cells and start *de novo* expressing CD95, which provides either co-stimulating or pro-apoptotic signals (Siegel et al., 2000). Other markers also appear during the activation process, the earliest activation markers (12 h) are CD69 and CD25, the α subunit of the IL2 receptor (Salgado et al., 2002). Expression of CD38 and HLA-DR is associated with late (1 day and 3–5 days, respectively) activation and proliferation of subset of mature T-cells (Amlot et al., 1996; Sandoval-Montes and Santos-Argumedo, 2005). CD134, also known as tumor necrosis factor receptor superfamily, member 4 (TNFRSF4 or OX40), is a specific marker of CD4^+^ T-cells activation, which increases their survival (Ladanyi et al., 2004). CD137, also called 4-1BB, stimulates survival and enhances the cytotoxic function of CD8^+^ T-cells which infiltrate the tumor (Zhu and Chen, 2014). Isolated from the tumor site CD137^+^ T cells can inhibit tumor cell growth *in vivo* (Ye et al., 2014).

T-cells can also differentiate into memory cells, which provide a quick immune response upon reinfection (Mahnke et al., 2013) and also contribute to the antitumor immunity. Traditionally, the classification into different memory populations takes place in the context of CD8^+^ T-cells. Memory cells can be located in the secondary lymphoid organs (central memory cells, T_CM_) or in recently infected tissues – effector memory cells, T_EM_ cells (Opata and Stephens, 2013). T_EM_ cells (CD45RA^–^, CD45RO^+^, CD62L^–^, CCR7^–^) provide a more rapid cytotoxic response during infections than T_CM_ (CD45RA^–^, CD45RO^+^, CD62L^+^, CCR7^+^) (Mahnke et al., 2013). However, in the context of a tumor, T_CM_ can more effectively suppress tumor growth compared to T_EM_ (Klebanoff et al., 2005). Another population of memory cells is resident memory T-cells (T_RM_) (CD103^+^, CD69^+^, CD49a^+^, CD62L^–^, CCR7^–^), which are located in various tissues of the body (Cheuk et al., 2017; Reading et al., 2018). Although their role in antitumor immunity is not fully understood, an increased number of CD8^+^ CD103^+^ T-cells are associated with prolonged patient survival (Dumauthioz et al., 2018). There are several different theories, which phenotype, effector or memory, appears first, the relationships between effector cells and memory cells which are comprehensively discussed in Restifo and Gattinoni (2013).

## NKT-Cells

Natural Killer T-cells are a group of cells that play an important role to link the innate and adaptive immunity since they have the characteristics of both conventional T-cells and NK cells (Kumar et al., 2017). NKT-cells mature in the thymus and acquire specific TCRs that can recognize lipid, but not protein, molecules represented by MHCI-like CD1d molecules (Metelitsa et al., 2001). Usually, NKT-cells are classified in two large groups depending on the structure of TCR chains and its ability to bind to lipid molecules. Type I NKT-cells bind to a common lipid prototype, α-galactosylceramide (α-GalCer) (Horikoshi et al., 2012). Since these cells express many typical NK cell and T-cell markers on their surface, fluorescent dye-conjugated α-GalCer lipid tetramers that will selectively bind to TCR are primarly used to detect NKT-cells (Kawano et al., 1997). Antibodies to specific fragments of TCR chains, such as Vα24 (especially clone 6B11) and Vβ11 are also used to identify these cells (Montoya et al., 2007). After interaction with α-GalCer type I NKT-cells activate (Kawano et al., 1997) and acquire typical activation markers, such as CD69 (Kitayama et al., 2016), CD38 (Chan et al., 2013), CD25 (Chan et al., 2013). Activated type I NKT-cells are capable of killing CD1d^+^ tumor cells in a CD1d-dependent manner (Ni et al., 2015). However, they will most likely mediate antitumor activity through activation of downstream immune effector cells. Secretion of large amounts of IFN-γ leads to the formation of tumor-specific CD8^+^ CTLs (Nishimura et al., 2000) and rapid activation of NK cells (Ishihara et al., 2000; Escriba-Garcia et al., 2017). A noteworthy detail is that type I NKT-cells express a variable number of CD4 and CD8 receptors depending on the donor. For example, neonatal NKT-cells are predominantly (>90%) CD4^+^, whereas adult peripheral blood NKT-cells are either CD4^+^ or CD4^–^. The same can be said about typical markers of NK cells, CD56 and CD161, which are expressed on NKT-cells in varying numbers. For example CD4^–^ cells express CD56 in 17–70% of cases and have a more significant cytotoxic function in the activated condition (Eger et al., 2006). However, the anti-cancer effect of the individual NKT-cell populations has not yet been investigated.

Another large group of NKT-cells is type II NKT-cells which do not have any specific receptors and do not respond to α-GalCer (Dasgupta and Kumar, 2016), but can recognize many other lipids, including sulfatides and lipopolysaccharides (LPS) (Chang et al., 2008; Blomqvist et al., 2009). For lack of any specific markers, it is sulfatide-loaded CD1d multimers with a fluorescent label that are often used to identify these cells (Zhang et al., 2011). The antitumor function of these cells is ambiguous, where most studies indicate that type II NKT-cells support tumor immunosurveillance by secreting IL13 (Terabe et al., 2003; Ambrosino et al., 2007; Fichtner-Feigl et al., 2008). However, Th1-like type II NKT cells are also able to suppress tumor growth due to IFN-γ secretion (Zhao et al., 2014).

In general, NKT-cells play an important role in the antitumor immune response, but their identification and clustering using surface markers is rather difficult due to small amount of cumulative knowledge.

## B Cells

Despite the fact a lot of attention is paid to the role of T-cells in the antitumor immune response, B cells play an equally important role in carcinogenesis and tumor progression (Tsou et al., 2016). B cells mature in the bone marrow and enter the peripheral blood as transitional B cells, which carry typical B cell markers CD19 and CD20 as well as are CD5^+^, CD38^high^, CD27^–^ (LeBien and Tedder, 2008). This population of B cells can differentiate into follicular (FO), marginal zone (MZ), and regulatory B cells (Bregs) (Clavarino et al., 2016). FO B cells (CD19^+^, CD20^+^, CD21^+^, CD22^+^, CD23^+^, CD24^+^, CD10^–^, CD27^–^) can activates after stimulation by antigen and differentiate into effector cells (Sarvaria et al., 2017). After binding to the antigen, B cells bind to Th1 cells via the MHC-peptide complex, B7 and CD40 on the surface of B cells (Watanabe et al., 2017). Such activated B cells (CD19^+^, CD20^+^, CD25^+^, CD27^+^, CD30^+^, CD69^+^, CD80^+^, CD88^+^) can become short-lived plasma cells (CD19^low^, CD20^–^, CD27^+^, CD38^high^, CD69^+^, CD138^+^), that secrete specific antibodies or form germinal centers (GCs) in lymph nodes and the spleen (De Silva and Klein, 2015). GCs are temporary formations in which activated B cells (CD10^+^, CD19^+^, CD20^+^, CD27^–^, CD33^+^, CD38^high^) continue their maturation and can develop into long-lived memory cells (CD19^+^, CD20^+^, CD27^+^, CD38^–^, CD40^+^, CD23^low^).

Another significant B cell population is MZ B cells (CD1c^+^, CD19^+^, CD20^–^, CD21^high^, CD27^*var*^), which recognize T-cell-independent carbohydrate and phospholipid antigens and produce multireactive IgM antibodies (Descatoire et al., 2014), providing a rapid response to T-cell- independent antigens.

Tumor-infiltrating B cells can have both pro- and antitumor effects (Yuen et al., 2016). B cells are capable of producing antibodies that target tumor intracellular antigens, for example, against aberrantly exposed β-actin (Hansen et al., 2001) or p53 (Kumar et al., 2009), the presence of the antibodies correlates with a favorable outcome of the disease. B cells can also act as APCs and stimulate the T-cell-mediated immune response (Bruno et al., 2017) or directly induce TRAIL/Apo-2L-mediated death of tumor cells (Kemp et al., 2004). However, B cells can also contribute to tumor progression due to the secretion of lymphotoxin (Ammirante et al., 2010) and Bregs, whose functions are discussed below.

The immunophenotype and functions of another B cell population have been studied more widely. Bregs seem to evolve from transitional B cells, however, it is suggested that mature B cells and plasmoblasts also have the ability to differentiate into IL10-producing Bregs (Rosser and Mauri, 2015). The function of IL10-producing CD1d^high^ CD5^+^ B cells, that suppress anti-tumor immunity by stimulating the development of Tregs (Liu and Zeng, 2012) and suppressing CD8^+^ T-cells is best described (Wei et al., 2016). The same properties to suppress CD4^+^ T-cell proliferation and effector function have been described for CD19^+^ CD24^high^ CD38^high^ Bregs (Bouaziz et al., 2010; Flores-Borja et al., 2013; Zhang et al., 2017). The various Bregs populations and their functions are described in Sarvaria et al. (2017). The role of B-cells in antitumor immunity is Janus-faced, as well as the one of T-cells, although they have not been investigated as intensively as T-cells. Apart from Bregs, pro- and antitumor properties are mainly determined for general CD19^+^ CD20^+^ B cell population, therefore, the role of each individual B cell population in the tumor immune response remains to be investigated.

## Dendritic Cells

The presentation of the antigen to the cells of an adaptive immunity is an essential for providing the antitumor immune response. Conventional population of DCs is one of the main sources of processed tumor antigens for T-cells (Gardner and Ruffell, 2016). DCs can be divided into two populations, DC1 and DC2, which develop depending on the activation of different transcription factors (Murphy et al., 2016). Both of these populations express CD11c and MHCII, with DC1 expressing X-C motif chemokine receptor 1 (XCR1) and dendritic cell NK lectin group receptor-1 [DNGR-1, also called C-type lectin domain family 9 member A (CLEC9A)] and CD141 (blood dendritic cell antigen 3, BDCA3) (Poulin et al., 2010). DC1 provide the processing of tumor antigens, migrate via CCR7 chemotaxis to the lymphoid organs and ensure antigen presentation to T-cells (Roberts et al., 2016). DC1 also express IFN-α receptor 1 (IFNAR1) because type I IFNs (IFN-α, IFN-β) promote their activation, migration and cross-presentation (Diamond et al., 2011). DC2 can be separated from DC1 by expression of CD11b, CD1c (BDCA1) and CD172a (Veglia and Gabrilovich, 2017), however, they are very difficult to distinguish from CD11c^+^ MHCII^+^ macrophages and monocyte-derived DCs (moDCs), so understanding the role of DC2 in tumor immunity remains limited.

A population of moDCs, also known as inflammatory DCs, are formed from CD14^high^ monocytes as a result of inflammation (Segura and Amigorena, 2013). These cells carry a large number of common markers of myeloid cells, namely CD1a, CD11c, BDCA1, CD172a, CD206, and HLA-DR (Veglia and Gabrilovich, 2017). However, they can be identified by the expression of CD64 or the receptor of granulocyte-macrophage colony-stimulating factor (GM-CSFR), since GM-CSF is necessary for the development of moDCs from monocytes (Hiasa et al., 2009). It is GM-CSF-generated moDCs that are used to create antitumor vaccines. *In vivo* differentiated moDC-based vaccines are safe but not effective in a large number of clinical trials in patients with various tumors (Anguille et al., 2014; Chulpanova et al., 2018a). The failure of moDCs-based vaccines to stimulate an antitumor immune response in patients may be due to the compromised functionality of immune cells isolated from cancer patients (Shinde et al., 2018). In the human body, moDCs are able to present antigen, which leads to the simulation of CD8^+^ T-cells, inhibition of tumor growth (Kuhn et al., 2015) and Th17 cell stimulation (Segura et al., 2013).

Another population with a described role in antitumor immunity is plasmacytoid DCs (pDCs), which are characterized by a high level of type I IFN secretion (Facchetti et al., 2003). These cells mature in the bone marrow and have a plasma cell-like morphology; typically, pDCs express CD4, HLA-DR, CD123, BDCA2 (CD303), BDCA4 (CD304) and do not express CD11c (Veglia and Gabrilovich, 2017). Through the secretion of IFN-α and other pro-inflammatory cytokines, pDCs promote innate immune responses via the induction of NK cell migration and stimulation of macrophages and dendritic cells (Mitchell et al., 2018). pDCs can also regulate the T-cell mediated immune response and act as APCs [for more details see Mitchell et al. (2018)]. Direct GrzmB and TRAIL-mediated antitumor activity of pDCs has also been reported in some studies (Tel et al., 2012; Lombardi et al., 2015). However, a number of studies of pDCs isolated from cancer patients show a tendency toward the formation of immunological tolerance of the tumor due to pDCs (Maldonado and von Andrian, 2010). TME appears to suppress IFN-α secretion in pDCs and stimulate the development of Tregs (Faith et al., 2007; Sisirak et al., 2013).

Markers of various populations of DCs and their role in the tumor immunity, which varies depending on the population, have been widely investigated. Antitumor properties of moDCs have been actively explored, however, the active use of DCs as a platform for the development of anticancer vaccines has not led to significant success in the treatment of cancer (Chulpanova et al., 2018c).

**FIGURE 1:**
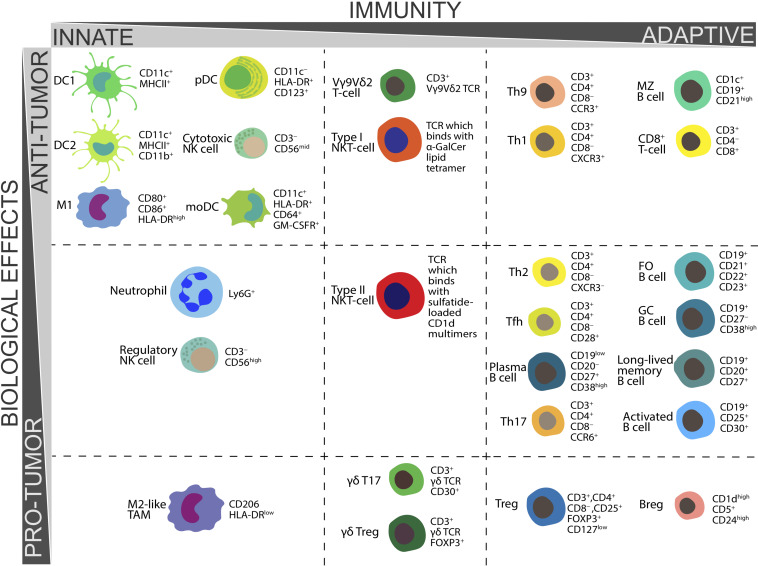
Biological functions in tumor immunity and CD markers essential for *ex vivo* determination of various populations of human immune cells.

The functions and CD markers of above populations of immune cells are summarized in Table 1 and Figure 1.

**TABLE 1:** Typical CD markers and functions in tumor immunity of various populations of human immune cells.

Immune cells		Typical CD markers	Anti-tumor properties	Pro-tumor properties
**NK cells**	**Cytotoxic**	**CD3**^–^, **CD56^mid^**, CD16^high^, NKp46, NKp30, NKG2D, KIRs	Perforin and granzyme-mediated induction of apoptosis	NA
	**Regulatory**	**CD3**^–^, **CD56^high^**, CD16^±^, NKp46, NKp30, NKG2D, KIRs	IFN-γ-mediated regulation of DC maturation	Pro-angiogenic effect
**Neutrophils**		CD11b^+^, CD66b^+^, CD16, **Ly6G**^+^, CXCR2^+^	Inhibition of tumor by IL1 and TNF-α secretion	Pro-angiogenic effect; suppression of CD8^+^ T-cell mediated immune response
**Macrophages**	**M1**	CD68^+^, **CD80**^+^, **CD86**^+^, CD206^low^, **HLA-DR^high^**	IL6, IL12, IL23 and TNF-α-mediated stimulation of immune response and reactive oxygen/nitrogen specie-mediated tumor killing	NA
	**M2-like TAMs**	CD163, CD200R, CD204, **CD206**, **HLA-DR^low^**	NA	VEGF-mediated ro-angiogenic effect; MMPs and cathepsin-mediated supports of invasion and metastasis; IL10, TGF-β, ARG1, and PGE-mediated suppression immune response
**γδ T-cells**	**Vγ 9Vδ 2 T-cells**	**CD3**^+^, **Vγ 9Vδ 2 TCR**, NKG2D^+^, CD56^+^, CD16^+^	TRAIL-induced apoptosis of tumor	NA
		**CD3**, **Vγ 9Vδ 2 TCR,** CD86^+^, CD80^+^, CD40^+^, MHCII^+^	Antigen-presenting cell-like activity	NA
	**γδ Tregs**	**CD3**^+^, **γδ TCR**, **FOXP3**^+^	NA	Suppression immune response
	**γδ T17**	**CD3**^+^, **γδ TCR**, **CD30**^+^	NA	Accumulation of immunosuppressive cells in TME; pro-angiogenic effect
**T-cells**	**Th1**	**CD3**^+^, **CD4**^+^, **CD8**^–^, **CXCR3**^+^, CCR4^–^, and CCR2^+^, CCR5^+^, CXCR6^+^ when activated	Stimulation of CD8^+^ T-cell expansion; IFN-γ-dependent inhibition of angiogenesis	NA
	**Th2**	**CD3**^+^, **CD4**^+^, **CD8**^–^, CCR4^+^, **CXCR3^–^**, CCR3, CCR8	IL4-mediated increase of tumor infiltration with eosinophils and macrophages	IL10-mediated inhibition of antigen presentation and activation of Tregs
	**Th17**	**CD3**^+^, **CD4**^+^, **CD8**^–^, **CCR6**^+^, CCR4^+^, CXCR3^+^, IL1R^+^, IL6R^+^, IL23R^+^	IL17-mediated recruitment of NK cells and neutrophils	IL17, IL23, CCL20-mediated promotion of tumor growth; IL17 and IL22 mediated suppression of CD8^+^ mediated immune response
	**Tfh**	**CD3**^+^, **CD4**, **CD8**^–^, **CD28**^+^, CD40L^+^, CXCR5^+^, ICOS^+^, PD-1^+^	Promotion of B cells differentiation; IL12-mediated stimulation of CD8 + T-cells	Can stimulate growth and survival of some lymphoid tumors
	**Tregs**	**CD3**^+^, **CD4**, **CD8**^–^, **CD25**^+^, **FOXP3**^+^, **CD127^low^**, PD-1^+^, CTLA-4^+^, CD39^+^, CD73^+^	NA	Suppression of CD8^+^ T-cell mediated immune response
	**Th9**	**CD3**^+^, **CD4**^+^, **CD8**^–^, **CCR3**^+^, CCR6^+^, CXCR3^+^, IL17BR	GrzmB-mediated tumor killing; TRAIL-mediated apoptosis induction; regulation of DCs and CD8^+^ T-cells	NA
	**CD8**^+^ **T-cells**	**CD3**, **CD4**^–^, **CD8**^+^, CD28^+^, CXCR3^+^, LFA-1^+^, CD25^+^, CD107a^+^, PD-1^+^, CTLA-4^+^	Granule exocytosis-mediated tumor killing and FasL-mediated apoptosis induction	NA
**NKT-cells**	Type I	**TCR which binds with α-GalCer lipid tetramer**, CD3, CD4^±^, CD8^±^, CD56^±^, CD161^±^	Direct killing CD1d^+^ tumor cells; IFN-γ-mediated stimulation of formation of tumor-specific CD8^+^ T-cells and activation of NK-cells	NA
	Type II	**TCR which binds with sulfatide-loaded CD1d multimers,** CD3^+^	IFN-γ-mediated suppression of tumor growth	IL13-mediated support of tumor immunosurveillance
**B cells**	**FO**	**CD19^+^**, CD20^+^, **CD21**^+^, **CD22**^+^, **CD23**, CD24^+^, CD10^–^, CD27^–^	Pro- and antitumor properties are mainly determined for general **CD19**^+^ **CD20**^+^ B cell population, the role of individual B cell population remains unclear Production of antibodies that target tumor intracellular antigens; antigen presentation and stimulation of T-cell-mediated immune response; TRAIL/Apo-2L-mediated induction of apoptosis	Stimulation of tumor progression due to the secretion of lymphotoxin
	**Activated**	**CD19**^+^, CD20, **CD25**^+^, CD27^+^, **CD30**^+^, CD69^+^, CD80^+^, CD88^+^		
	**Short-lived plasma cells**	**CD19^low^**, **CD20**^–^, CD27^+^, **CD38^high^**, CD69^+^, CD138^+^		
	**GC cells**	CD10^+^, **CD19**^+^, CD20^+^, **CD27**^–^, CD33^+^, **CD38^high^**		
	**Long-lived memory cells**	**CD19**^+^, **CD20**^+^, **CD27**^+^, CD38^–^, CD40^+^, CD23^low^		
	**MZ**	**CD1c**^+^, **CD19**^+^, CD20^–^, **CD21^high^**, CD27^*var*^	Recognize T-cell-independent carbohydrate and phospholipid antigens	NA
	**Bregs**	**CD1d^high^ CD5**^+^, CD19^+^, **CD24^high^**, CD38^high^	NA	IL10-mediated suppression of CD8^+^ T-cell mediated immune response and stimulation of development of Tregs
**DCs**	**DC1**	**CD11c**^+^, **MHCII**^+^, XCR1^+^, CLEC9A^+^, CD141^+^, IFNAR1^+^	Antigen presentation to T-cells	NA
	**DC2**	**CD11c**^+^, **MHCII**^+^, **CD11b**^+^, CD1c^+^, CD172a^+^	The function is not clear since they are very difficult to distinguish from CD11c^+^ MHCII^+^ macrophages moDCs	NA
	**moDCs**	**CD11c**^+^, **HLA-DR**^+^, **CD64**^+^, **GM-CSFR**^+^, CD1c^+^, CD1a^+^, CD172a^+^, CD206^+^,	Presentation of antigen which leads to the simulation of CD8^+^ T-cells, inhibition of tumor growth and Th17 cell stimulation	NA
	**pDCs**	**CD11c**^–^, **HLA-DR**^+^, **CD123**^+^, CD4^+^, BDCA2 (CD303)^+^, BDCA4 (CD304)^+^	Induction of NK cell migration and stimulation of macrophages and dendritic cells; GrzmB and TRAIL-mediated apoptosis induction	NA

*Surface markers essential for flow cytometry-based determination of immune cells are in bold.*

## Molecular Aspects of Cytokine-Based Cancer Therapy

### Interleukin 2

The history of the use of cytokines as agents for the treatment of various diseases, including cancer, began in the mid-1990s when the anticancer effect of High-dose (HD) IL2 therapy was first demonstrated (West, 1989). IL2 is predominantly produced by antigen-stimulated CD4^+^ T-cells (Choudhry et al., 2018), as well as NKT-cells, CD8^+^ T-cells, mast cells and DCs (Paliard et al., 1988; Granucci et al., 2001; Yui et al., 2004; Hershko et al., 2011). IL2 can stimulate the proliferation of antigen-activated CD8^+^ T-cells (Cornish et al., 2006), treatment with endogenous IL2 leads to an increase in the expression of CD25, IL2 receptor, which in turn stimulates the proliferation of CD8^+^ T-cells (Hinrichs et al., 2008). IL2 increases the expression of LAMP-1 on the surface of CD8^+^ T-cells (Hromadnikova et al., 2016), decreases the expression of PD-1, an immunosuppressive receptor (Thommen et al., 2018), thereby mediating the cytotoxic activity of CD8^+^ T-cells. IL2 can stimulate the expansion and activation of NK cells with CD56^high^ CD16^–^ receptor (Caligiuri et al., 1993), with the cytotoxic function of this population is increased after activation (Fehniger et al., 2003). This cytokine also enhances the cytotoxic effect of γδ T-cells, increasing CD69 and degranulation marker CD107a expression and IFN-γ secretion (Ribot et al., 2014). Furthermore, IL2 can also promote the expansion of NKT-cells in cancer patients (Euhus et al., 1997; Engel et al., 1998). IL2-based therapy has several significant drawbacks, particularly the stimulation of Tregs, which are associated with the suppression of the antitumor immune response (Boyman et al., 2006). Stimulation with IL2 leads to increased expression of CD25, CTLA-4, and HLA-DR (Hirakawa et al., 2016) on the surface of CD3^+^ T-cells. IL2 treatment leads to an increase in the expression of FOXP3 in CD3^+^CD25^+^ T-cells and the formation of the Tregs phenotype, including during low-dose therapy (Zorn et al., 2006). However, recent discoveries in understanding the functioning of Tregs have opened up new possibilities for the use of IL2 in combination with Treg inhibitors, such as anti-CTLA-4 and anti-PD-1 (West et al., 2013). The combination of IL12 (Prochazkova et al., 2012) and IL21 (Attridge et al., 2012) has also been shown to block IL2-induced Treg activation.

Currently, IL2 has found its place in cancer immunotherapy for the expansion of immune cells such as NK cells, T-cells, NKT-cells, cytokine-induced killer (CIK) cells (Chung et al., 2014; Boyiadzis et al., 2017; Exley et al., 2017; Yoshida et al., 2017). IL2 is also used as an adjuvant in the treatment of patients with melanoma, advanced colorectal cancer or ovarian cancer with autologous dendritic cells stimulated by autologous tumor lysate (Baek et al., 2015; Greene et al., 2016; Liu et al., 2016), as well as in the treatment with viral vaccines (Oudard et al., 2011). The addition of IL2 to the treatment regimen can increase the effectiveness of therapy due to the induction of expansion of tumor antigen presented T-cell (Oudard et al., 2011).

Interleukin 2 is also effective to treat patients with melanoma when combined with dacarbazine monotherapy or anti-VEGF monoclonal antibody (mAb) monotherapy, but do not increase the effectiveness of neuroblastoma therapy in combination with dinutuximab (anti-GD2 mAb) (Ladenstein et al., 2018; Tarhini et al., 2018; Weide et al., 2019). Several clinical trials have also evaluated the effectiveness of the combination of radiotherapy and IL2, however, this approach has not been further developed (Oudard et al., 2011; Ridolfi et al., 2014; van den Heuvel et al., 2015).

One of the newest approaches to cancer immunotherapy is the combination of immune checkpoint inhibitors and recombinant IL2 for the treatment of melanoma and renal cell carcinoma. Such a combination may improve the activation of the immune system (West et al., 2013) and potentially enhance clinical efficacy. HD IL2 increased overall survival (OS) in patients with renal cell carcinoma and progressive melanoma who was previously treated with a PD-1 or PD-L1 inhibitor compared to patients without pretreatment with anti-PD-1/PD-L1 (Buchbinder et al., 2019). The combination of IL2 and ipilimumab (CTLA-4 checkpoint inhibitor) for the treatment of patients with melanoma led to large side effects expected from single agent treatment, with an increase in the number of peripheral IFN-γ producing CD8^+^ T-cells in most patients, which indicated the effectiveness of IL2 and ipilimumab combination to stimulate the immune response (Ray et al., 2016; Weide et al., 2017; Silk et al., 2019).

### Interleukin 12

Interleukin 12 is another cytokine with well-studied antitumor activity, which is mainly mediated by stimulation of IFN-γ production in cytotoxic cells (CD8 T-cells and NK cells) and Th1 cells (Lasek et al., 2014). Treatment with IL12 leads with varying degrees of success to an increase in the number of CD56^+^ NK cells (Agaugue et al., 2008), as well as CD2 and LFA-1 expression (Robertson et al., 1999), which ultimately results in the increased cytotoxicity of IL12-treated NK cells (Lehmann et al., 2014; Martinovic et al., 2015). IL12 also stimulates the production of IFN-γ in cytotoxic CD8^+^ T-cells (Yang et al., 2016), enhances their proliferative activity (Mukhopadhyay et al., 2019) and cytotoxicity, probably due to increased expression of GrzmB (Rubinstein et al., 2015; Li et al., 2017). In addition, IL12-stimulated CD8 T-cells are able to reduce the number of Tregs in TME by Fas-mediated apoptosis (Kilinc et al., 2009; Kerkar et al., 2010). IL12 is also involved in the differentiation of naïve Th cells in Th1 cells (Kennedy et al., 1994). Promising results in cell and animal models have prompted clinical studies of IL12. However, the future of IL12 in the treatment of cancer was overshadowed by the significant side effects that were observed in the first clinical trials. The established protocol in the Phase I study schedule of IL12 administration was slightly amended (patients in Phase 2 trial received daily IL12 dosing without the single injection of IL12 2 weeks earlier that was employed in the Phase 1 study) that led to the development of severe IFN-γ-mediated toxicity, resulting in 12 patients being hospitalized and two patients dying (Leonard et al., 1997). Clinical trials of IL12 have continued, but with more caution, however, ultimately IL12 has not been shown particular efficacy, either alone or in combination with various therapeutic agents, with the exception of patients with cutaneous T-cell lymphoma (CTCL), with non-Hodgkin’s B-cell lymphoma, and with acquired immune deficiency syndrome (AIDS)-associated Kaposi sarcoma (Lasek et al., 2014).

To avoid the high IL12-mediated systemic toxicity, regulated plasmids have been developed in which IL12 expression is induced by an activator. The use of a plasmid in which the expression of IL12 is regulated by a promoter sensitive to nuclear factor of activated T-cells (NFAT) for the genetic modification of TILs has allowed a reduction in the number of cells necessary for significant response by 10–100 times in comparison with standard treatment protocols. However, serum IL12 levels were unpredictable and significant toxicity was observed in patients (Zhang et al., 2015). In another study, a regulated plasmid was injected at the site of tumor resection, and IL12 expression was then activated. After this therapy OS rates were encouraged compared to historical controls (Chiocca et al., 2019).

In combination with chemotherapy (Anwer et al., 2013) or mAbs (McMichael et al., 2019) IL12 did not show significant toxicity, as well as promising result indicating the usefulness of IL12.

An actively developing approach is the use of IL12 in combination with oncolytic viruses (OVs), which selectively replicate in and kill cancer cells. The integration of the *IL12* gene in the virus genome can enhance the virus-mediated immune response, while avoiding systemic toxicity (Nguyen et al., 2020). OVs with IL12 showed safety and promising efficacy in mouse models compared with OS without IL12 (Cheema et al., 2013; Alessandrini et al., 2019), however, the results of clinical trials on humans have not yet been presented (Patel et al., 2016) (NCT02555397, NCT00406939, NCT03281382, NCT00849459, and NCT01397708).

### Interleukin 15

Another cytokine that belongs to the same family as IL2 and has many overlapping functions is IL15 (Waldmann, 2018). Since IL15 and IL2 use several identical receptor components, IL2/IL15Rβ and γc, and trigger the common molecules of Janus kinase (JAK) 1/3 and signal transducer and activator of transcription (STAT) 3/5 pathways they have overlapping functions (Robinson and Schluns, 2017). The administration of IL15 leads to an increase in the expansion of CD8^+^ T-cell, as well as expression of CD38 and HLA-DR activation markers (Conlon et al., 2015). This cytokine also enhances the proliferation of NK cells in cancer patients with greater stimulation of CD56^high^ NK cells (Dubois et al., 2017). IL15 can also stimulate the cytotoxic functions of CD56^high^ NK cells (Wagner et al., 2017), while increasing the expression of CD16 and CX3C chemokine receptor 1 (CX3CR1), which are mainly expressed on CD56^low^ NK cells (Dubois et al., 2017). A similar effect (enhanced expansion and cytotoxicity) IL15 has on γδ T-cells (Ribot et al., 2014; Conlon et al., 2015). Despite similar functions with IL2, IL15 therapy does not stimulate the expansion of Tregs and does not cause capillary leak syndrome. However, recombinant IL15 therapy can lead to general systemic toxicity by increasing the production of pro-inflammatory cytokines or by stimulating autoimmune-like responses (Cooley et al., 2019).

Phase I clinical trials of subcutaneous recombinant human IL15 (rhIL15) therapy showed that rhIL15 was well tolerated and caused expansion of circulating NK cells, especially CD56^bright^ subset, and to a lesser degree CD8^+^ T-cells (Miller et al., 2018). Since IL15 is *trans*-presented to CD8^+^ T and NK cells in a complex with the IL15α receptor in order to increase its immunomodulating activity, the ALT-803 therapeutic complex has been developed, which is IL15 with IL15α receptor fused to human IgG1 dimer Fc, which maintains stability and extends the half-life of the entire complex (Knudson et al., 2019). ALT-803 showed safety and the ability to increase the number of NK cells after subcutaneous administration to patients with progressive solid tumors in phase I clinical trials (Margolin et al., 2018). Clinical effect demonstration in single-agent phase I trials is rare, and has not been observed in these trials. However, several clinical trials to evaluate the therapeutic potential of rhIL15 in combination with other therapeutic agents are ongoing (NCT03905135, NCT03388632).

ALT-803 in combination with nivolumab (anti-PD-1 mAb) may repeatedly elicit objective responses to anti-PD-1 immunotherapy after relapse or treatment failure in patients with non-small cell lung cancer (NSCLC) (Wrangle et al., 2018).

Interleukin 15 is actively used to activate and expand NK cells, which are then transplanted to patients with leukemia (Vela et al., 2018) or solid tumors (Perez-Martinez et al., 2015), as well as to stimulate the proliferation of chimeric antigen receptor (CAR) T-cells in combination with IL2 (Wang et al., 2019). In a recent clinical trial, anti-CD19 CAR-NK cells encoding the human IL15 gene were used to treat patients with recurrent or refractory CD19-positive cancer. Most patients had a response to treatment with CAR-NK cells without the development of major toxic effects (Liu et al., 2020).

Mouse models also showed the efficacy of using IL15/IL15R in combination with an autologous vaccine to stimulate antitumor immunity against acute myeloid leukemia (Shi Y. et al., 2018). However, these studies have not yet reached human clinical trials. It is most likely that in the near future combinations of IL15 with various therapeutic agents, such as immune checkpoint inhibitors, will be actively investigated.

### Interleukin 21

Interleukin 21, another member of the IL2 family, is one of the last cytokines investigated for the clinical use in cancer treatment (Santegoets et al., 2013). One of the most important functions of IL21 is to stimulate the proliferation of germinal center (GC) B cells (Zotos et al., 2010), as well as to induce the differentiation CD40L-stimulated B cells in plasma cells (Ding et al., 2013). However, IL21 treatment also results in an increase in the number of B10 cells as well as the levels of IL10 that they produce (Yoshizaki et al., 2012). The increase in IL10 secretion after IL21 stimulation is also observed in CD4^+^, CD8^+^ T-cells (Spolski et al., 2009). IL21 is also able to activate NK cells by stimulating the expression of CD69 and the natural cytotoxicity receptor NKp46 and increasing cytotoxic activity (Skak et al., 2008). IL21 stimulates differentiation of naïve CD4^+^ T-cells in Th17 cells, inducing expression of IL17, retinoic-acid-receptor-related orphan nuclear receptor gamma (RORγt) transcription factor and IL23R (Korn et al., 2007). Also, this cytokine plays an important role in the autocrine stimulation of proliferation and differentiation (expression of inducible T-cell costimulator (ICOS) is increased) of Tfh (Vogelzang et al., 2008). It is also worth noting that IL21 negatively regulates homeostasis of CD4^+^ CD25^+^ FOXP3^+^ Tregs (Attridge et al., 2012). However, in clinical trials the therapeutic effect of IL21 is evaluated by analyzing the secretion of soluble CD25, the level of which increases during the activation of T-cells and NK cells (Thompson et al., 2008; Davis et al., 2009). Whilst the effect of IL21 therapy on the remaining immune system cell populations of cancer patients remains unexplored.

Clinical trials have shown that rIL21 is able to increase the number of CD3^+^ CD56 NKT-like cells in patients with stage IV malignant melanoma (Coquet et al., 2013), and also activate T and NK cells in patients with stage IV colorectal cancer (Steele et al., 2012). The combination of IL21 with various mAbs showed that the combination of IL21 with rituximab (anti-CD20 mAb) or sorafenib (anti-VEGF mAb) was well tolerated and had antitumor activity (Timmerman et al., 2012; Bhatia et al., 2014). However, the contribution of IL21 into the shown antitumor activity has not been fully determined.

Interleukin 21, like other members of its family, is used to activate and expand T-cell and NK cells (Chapuis et al., 2016; Ciurea et al., 2017). There are several completed and active clinical trials where IL21 is combined with checkpoint inhibitors or with anti-CD19 CAR T-cells (NCT01629758, NCT04093648). There is insufficient clinical data on the use of this cytokine. However, it is likely that the use of IL21 in cancer immunotherapy will be investigated in this direction.

### Interferons

Another group of cytokines, interferons, has also been shown to be effective in cancer immunotherapy. IFNs are divided into three types depending on the function and the target receptor: type I (α, β, ε, κ, and ω), type II (γ), and type III (λ) (Budhwani et al., 2018). IFN-α2a was the first approved cytokine for the treatment of chronic myeloid leukemia (Italian Cooperative Study Group on Chronic Myeloid Leukemia, Tura et al., 1994), since type I IFNs have broad immunomodulatory activity. IFN-α can stimulate differentiation of CD14^+^ monocytes in DCs jointly with GM-CSF (Gabriele et al., 2004) which will be discussed below. IFN-α/GM-CSF stimulation leads to the increased expression of HLA-DR, CD11c, CD83, B7 costimulatory molecules CD80 and CD86, including on DCs of cancer patients, and such DCs are able to efficiently present an antigen to CD4^+^ and CD8^+^ T-cells (Paquette et al., 2002; Jin et al., 2017). However, IFN-β reduces the ability of mature DCs to stimulate T-cell proliferation and differentiate into IFN-γ-producing Th1 cells (Yen et al., 2010). In addition to its apparent effect on T-cells via DCs, IFN-α is also able to stimulate the effector functions of pre-activated CD8^+^ T-cell (which have already interacted with antigen and costimulatory molecules), leading to increased number of activation markers CD38 and CD25 and raised expression of GrzmB, TRAIL, FasL, and IFN-γ (Sikora et al., 2009; Hervas-Stubbs et al., 2010; Lu et al., 2019). Type I IFNs have different effects on the differentiation of CD4^+^ T-cells, supporting the polarization into Th1 cells and inhibiting the formation of Th2 and Th17 T-cell phenotypes (Huber et al., 2010; Huber and Farrar, 2011). Type I IFNs also play an important role in the regulation of NK cell cytotoxicity, however, low cytotoxicity in the absence of type I IFN stimulation can be overcomed by stimulation with IL2 (Muller et al., 2017).

IFN-γ is a type II IFN cytokine which can both regulate the antitumor immune response and directly induce apoptosis of tumor cells (Zaidi, 2019). Here we focus on the regulation of the immune system cells. It was shown that IFN-γ therapy leads to a significant increase in the number of CD14^high^ CD16^+^ monocytes and a rise in MHCII expression on all monocytes (Kirkwood et al., 1997) in patients with various types of tumors. The number of activated NK cells also increases (expression of NK receptor activation marker NKp30 was increased on both CD56^high^ and CD56^low^ NK cells) (Zibelman et al., 2017) (NCT02614456). In addition, a number of investigations in mouse models has been shown that IFN-γ is able to modulate polarization toward CD86^+^ iNOS^+^ M1 macrophages, which can inhibit tumor cell growth by releasing NO (Ren et al., 2014; Muller et al., 2018). Nevertheless, IFN-γ stimulates differentiation of CD4^+^CD25^–^ T-cells in CD4^+^ Tregs in a mouse model of experimental autoimmune encephalomyelitis (Wang et al., 2006) as well inhibiting the proliferation of Th2 cells (Gajewski and Fitch, 1988; Oriss et al., 1997). However, the effect of IFN-γ on various T-cell populations in cancer patients requires more detailed investigation. In addition, there is an evidence of the pro-tumor effect of IFN-γ [for more details see Zaidi (2019)] which also requires the attention of researchers.

Type I interferons are actively combined with various therapeutic agents in clinical trials (NCT03112590). In the number of clinical trials, IFN-α or IFN-β were used to stimulate the immune response in patients who received therapy with DC vaccines (Schwaab et al., 2009; Duggan et al., 2016) or tumor-specific antigens (Elkord et al., 2015; Hawkins et al., 2016; Shima et al., 2019). Vaccination itself did not always lead to the increase in OS or showed some encouraging results (Shima et al., 2019). However, IFN-α administration was sometimes able to significantly increase OS compared to a single vaccine (Duggan et al., 2016; Sheng et al., 2020).

Interferons have also been combined with chemotherapy and mAbs for the treatment of various types of tumors (Dijkgraaf et al., 2015). The combination of IFN-β with temozolomide did not show any promising results in patients with glioblastoma (Wakabayashi et al., 2018). Also, the combination of IFNs with bevacizumab (anti-VEGF mAb) did not show much benefit compared to the combination of bevacizumab + everolimus, which is usually used to treat metastatic renal cell carcinoma (Ravaud et al., 2015).

In general, there are not many clinical trials that are ongoing to evaluate the efficacy of combination of IFNs with immune checkpoint inhibitors, most often IFN-α monotherapy is used as a reference to evaluate the effectiveness of the inhibitors for the treatment of advanced melanoma (Li et al., 2020; Tarhini et al., 2020). However, the clinical trials of IFN-α and pembrolizumab have shown the safety but had limited antitumor activity of the IFN-α + pembrolizumab combination (Atkins et al., 2018). Several clinical trials of IFN-γ with PD-1 inhibitors are ongoing (NCT02614456, NCT03063632).

### Granulocyte-Macrophage Colony-Stimulating Factor

Granulocyte-Macrophage Colony-Stimulating Factor plays an important role in the regulation of proliferation and differentiation of myeloid cells (Bhattacharya et al., 2015). One of the main functions of GM-CSF that is actively used for cancer immunotherapy is its ability to regulate the maturation of DCs from myeloid progenitors (Ushach and Zlotnik, 2016). Stimulation of CD14^high^ or CD133^+^ monocytes with GM-CSF together with IL4 and/or type I IFNs leads to differentiation in moDCs expressing MHCII, CD80, CD83, and CD86 costimulatory molecules (Moldenhauer et al., 2010; Blyszczuk et al., 2013). This cytokine has been used in a large number of clinical trials of antitumor vaccines (Chang et al., 2000; Slingluff et al., 2003). However, such moDCs are able to stimulate Th17 due to the secretion of IL1b and IL6, as already noted above (Ko et al., 2014), and also provoke the formation of FOXP3^+^ Tregs (Gopisetty et al., 2013). GM-CSF is also involved in the programming of M1 macrophages, and may promote M2 to CD45^+^ CD11b^+^ F4/80 MHCII^+^ CD163^–^ CD206^–^ M1 polarization of macrophages in TME (Eubank et al., 2009; Brenot et al., 2018; Benner et al., 2019). The ability of GM-CSF to stimulate the proliferation of activated CD54^+^ neutrophils has also been described, which may be useful for immunotherapy, since neutropenia is one of the most common symptoms of cytokine-based immunotherapy in cancer patients (Yu and Hua, 2018). However, the effect of neutrophils on tumor progression is still under discussion, and GM-CSF can stimulate the expression of PD-L1, an immunosuppressive molecule, on the surface of neutrophils (Wang et al., 2017). Despite the pronounced stimulation of differentiation of DCs, the effect of GM-CSF on other populations of the innate immune system cells remains insufficiently explored, especially in cancer patients. The use of this cytokine requires broader inquiry, because, despite active investigations, GM-CSF-based therapeutic vaccines have not shown the expected effectiveness.

Despite this, the number of clinical trials of antitumor vaccines that are used in combination with GM-CSF remains unchanged. Over the past 5 years (2015–2020), 58 new clinical trials have been registered, whilst between 2010 and 2015 69 clinical trials of antitumor vaccines were registered^1^. GM-CSF is added as an adjuvant to DCs for both DC-based vaccines and autologous tumor cell vaccines (Dillman et al., 2018; Clifton et al., 2019). Tumor cells can also be modified to overexpress GM-CSF to generate the vaccines (Gray et al., 2018). Interestingly, the administration of GM-CSF led to an increase in anti-GM-CSF neutralizing antibodies (Nabs), which, however, correlated with improved relapse-free survival (RFS) and OS (Butterfield et al., 2017).

Granulocyte-Macrophage Colony-Stimulating Factor was combined with radiation therapy to stimulate the maturation of DCs that could present antigens released by radiation-damaged cells. The combined therapy caused objective abscopal responses in some patients with metastatic solid tumors (Golden et al., 2015). The efficacy of T-vec oncolytic virus encoding the GM-CSF gene for the treatment of patients with melanoma was also shown (Andtbacka et al., 2016).

As with other cytokines, there is a tendency to combine cell-based and DNA-based vaccines + GM-CSF with immune checkpoint inhibitors (NCT04013672, NCT03600350) in order to achieve a significant therapeutic effect. In clinical trials, immune checkpoint inhibitors are combined with oncolytic viruses containing GM-CSF (NCT02977156, NCT04197882, NCT03206073, and NCT03003676). All of these clinical trials are now ongoing.

The effect of cytokine therapy on various populations of immune cells is summarized in Table 2.

**TABLE 2:** The effect of endogenous cytokines on various populations of immune cells.

Immune cells	Cytokines						
	
	IL2	IL12	IL15	IL21	Type I IFNs	IFN-γ	GM-CSF
**Innate immunity cells: NK cells, Neutrophils**, **Macrophages, DCs**	**NK cells:** CD56, proliferation, cytotoxicity↑	**NK cells:** CD2, LFA-1, proliferation, cytotoxicity↑	**NK cells:** CD16, CX3CR, proliferation, cytotoxicity↑	**NK cells:** CD69, NKp46, proliferation, cytotoxicity↑	**NK cells:** Cytotoxicity↑ **DCs:** IFN-α: differentiation in moDCs, HLA-DR, B7, CD11c, CD80, CD83 and CD86↑ IFN-β: ability of mature DCs to stimulate T-cell proliferation and differentiate into Th1↓	**NK cells:** NKp30, proliferation↑ **Macrophages:** Number of CD14^high^ CD16^+^ monocytes; M1 polarization↑	**Neutrophils:** PD-L1, proliferation↑ **Macrophages:** M1 polarization↑ **DCs:** Differentiation in moDCs, MHCII, CD80, CD83, CD86↑
**NKT-cells, γδ T-cells**	**NKT-cells**: Proliferation↑ **γδ T-cells**: CD69, CD107a, IFN-γ, cytotoxicity↑	NA	**NKT-cells**: Proliferation, cytotoxicity↑	NA	NA	NA	NA
**Adoptive immunity cells: Th1, Th2, Th17, Tfh, Tregs, CD8**^+^ **T-cells, FO B cells, Bregs**	**Tregs:** CD25, CTLA4, and HLA-DR, FOXP3, proliferation↑ **CD8**^+^ **T-cells**: CD25, LAMP-1, proliferation, cytotoxicity↑ PD-1↓	**Th1**: IFN-γ, differentiation from naïve T-cells↑ **Tregs:** The number in TME↓ **CD8**^+^ **T-cells**: IFN-γ, GrzmB, proliferation, cytotoxicity↑	**Tregs:** No effect **CD8**^+^ **T-cells**: CD38, HLA-DR, proliferation, cytotoxicity↑	**Th17**: Differentiation from naïve T-cells, IL17, RORγt, IL23R↑ **Tfh:** Proliferation, differentiation, ICOS↑ **Tregs:** Proliferation↓ **FO B cells:** GC B cell proliferation, plasma cell differentiation↑ **Bregs:** B10 cell proliferation, IL10↑	**Th1**: Differentiation↑ **Th2**: Differentiation↓ **Th17**: Differentiation↓ **CD8**^+^ **T-cells**: CD38, CD25, GrzmB, TRAIL, FasL, IFN-γ, cytotoxicity↑	**Th2**: Proliferation↓ **Tregs:** Proliferation↑	NA

*↑ – increase in expression/secretion or some function (activity), ↓ – decrease in expression/secretion or some function (activity).*

## Conclusion and Future Perspectives

Undoubtedly, cytokines have proven to be effective in cancer therapy, however, the effect of some promising targets on various immune cell populations remains poorly understood. The same situation is with respect to surface CD markers. For well-studied populations, such as T-cells, the set and functions of surface receptors are fairly well defined, and researchers use approximately the same sets of CD markers to identify populations using flow cytometry. However, less studied are the population of immune cells, the more diverse are the sets of determined receptors, and the more difficult it is to compare the results to identify persistent patterns. The study of changes in the function and surface marker expression of each individual immune system cell population after immunotherapy will simplify and unify the assessment of the effectiveness of therapy, as well as allow predicting the effectiveness of immunotherapy by analyzing surface markers of immune cells of cancer patients.

Despite the fact that this review focuses on a detailed description of the functions and surface markers of immune system cells in cancer and after cytokine-based immunotherapy, a review of this topic requires discussion of the prospects of cytokine-based cancer treatment. One of the main features of cytokines, as regulators of the immune response, is its pleiotropic effect. Each cytokine regulates many different populations of the immune cells that can support both anti-tumor and pro-tumor responses. Therefore, the future perspectives of cytokine-based cancer therapy will depend on the production of combined schemes aimed at enhancing the antitumor response and suppressing immune cells that support tumor growth. Also, other significant problems encountered are the short half-life and systemic toxicity (pro-inflammatory and autoimmune reactions) of high doses of cytokines which are necessary to elicit a significant response in cancer patients. New approaches that improve targeting of cytokines and alter their pharmacokinetics might be useful (such cell based or other vector delivery, chemically modified recombinant proteins, etc.) to overcome limitations of different cytokine therapies. Current trends in the development of cancer immunotherapy indicate that cytokines may find their greatest role in therapy when administered in combination with other agents, such as immune checkpoint inhibitors, oncolytic viruses or as a component of DC-based and tumor cell-based vaccines.

## Author Contributions

DC wrote the manuscript and made the tables. KK created the figure. AG edited the manuscript. DC, VS, and AR conceived the idea and edited the manuscript and tables.

## Conflict of Interest

The authors declare that the research was conducted in the absence of any commercial or financial relationships that could be construed as a potential conflict of interest.
